# Analysis of social metrics on scientific production in the field of emotion-aware education through artificial intelligence

**DOI:** 10.3389/frai.2024.1401162

**Published:** 2024-04-08

**Authors:** Jacobo Roda-Segarra, Santiago Mengual-Andrés, Andrés Payà Rico

**Affiliations:** Department of Comparative Education and History of Education, University of Valencia, Valencia, Spain

**Keywords:** artificial intelligence, emotion-aware, education, social impact, social media

## Abstract

Research in the field of Artificial Intelligence applied to emotions in the educational context has experienced significant growth in recent years. However, despite the field’s profound implications for the educational community, the social impact of this scientific production on digital social media remains unclear. To address this question, the present research has been proposed, aiming to analyze the social impact of scientific production on the use of Artificial Intelligence for emotions in the educational context. For this purpose, a sample of 243 scientific publications indexed in Scopus and Web of Science has been selected, from which a second sample of 6,094 social impact records has been extracted from Altmetric, Crossref, and PlumX databases. A dual analysis has been conducted using specially designed software: on one hand, the scientific sample has been analyzed from a bibliometric perspective, and on the other hand, the social impact records have been studied. Comparative analysis based on the two dimensions, scientific and social, has focused on the evolution of scientific production with its corresponding social impact, sources, impact, and content analysis. The results indicate that scientific publications have had a high social impact (with an average of 25.08 social impact records per publication), with a significant increase in research interest starting from 2019, likely driven by the emotional implications of measures taken to curb the COVID-19 pandemic. Furthermore, a lack of alignment has been identified between articles with the highest scientific impact and those with the highest social impact, as well as a lack of alignment in the most commonly used terms from both scientific and social perspectives, a significant variability in the lag in months for scientific research to make an impact on social media, and the fact that the social impact of the research did not emerge from the interest of Twitter users unaffiliated with the research, but rather from the authors, publishers, or scientific institutions. The proposed comparative methodology can be applied to any field of study, making it a useful tool given that current trends in accreditation agencies propose the analysis of the repercussion of scientific research in social media.

## 1 Introduction

Despite the numerous definitions of Artificial Intelligence (AI), this research will adhere to Dobrev’s (2012) definition, which posits AI as software that, by simplifying the world and based on stimuli, would provide responses at least similar to those a person would give. This definition emphasizes the comparative aspect between machines and humans, a key element inherent in the definition of this technology (human intelligence emulated by artificial machinery).

This was the approach proposed by Turing, a pioneer in computer science in the early 20th century, when he introduced his famous Turing test, reducing the philosophical question surrounding machines’ potential to think to a more empirical matter: it did not matter whether they actually think or not, but whether they seem to. Turing thus reduced AI to an imitative question (Mira et al., 2003). Turing also contributed his conceptual model of the Turing machine, which was a virtual machine that could be programmed to operate in a certain way and solve a specific problem. This virtual machine consisted of a hypothetical infinite tape where both data and instructions could be stored, a head that moved the tape and could read and write data at specific positions on the tape, and a control unit that allowed this virtual machine to be in a particular state at a given time (Brookshear, 1993). Based on this theoretical framework, Turing defined his Universal Turing Machine, a much more generic concept consisting of a Turing machine that could behave like other Turing machines based on the instructions encoded on the tape. In other words, a generic machine that could function as any other machine depending on the instructions contained on the tape. This laid the foundation for current microprocessors, which are nothing more than generic machines that, based on the instructions contained in a program, can operate in different ways. In the Universal Turing Machine, we find the foundations of computing and, by extension, the ability of modern AIs to mimic some human decision-making.

This concept of AI from an imitative perspective provides a broad framework that encompasses many other possibilities of this technology, such as identification, classification, or prediction. From this imitative perspective, AI would be software capable of performing identification, classification, or prediction tasks no worse than a person (Dobrev, 2012). These classificatory and predictive/identificatory possibilities of AI have been widely used in education for tasks such as academic performance classification or predicting school dropout (Gil et al., 2018; Castrillón et al., 2020; Mourdi et al., 2020; Landa et al., 2021; Jokhan et al., 2022).

Current research in AI stems from the connectionist branch of the discipline, with a different focus from the symbolic approach that had its heyday in the 1970s and fueled expert systems and knowledge-based systems. For the connectionist approach, knowledge resides in the structure of the network itself, which is self-programmable through learning. Mira et al. (2003) assert that the necessary condition to affirm that a software is AI is that some form of learning must occur.

For AI to have this self-programming capability, it requires algorithms, which are abstract machines (Moschovakis, 2001) that follow sequential steps to produce results based on input parameters. Similarly, but in a more generic sense, Knuth (1997) defines an algorithm as a finite set of rules aimed at solving a specific problem. Algorithms can be understood as the conceptual machines of Turing as previously discussed, and like any other computational process, they have the upper limit of their power level constrained by that of a Turing machine.

These algorithms are what nourish AI in its ability to reorganize and reprogram its own internal structure, enabling the resolution of problems that do not have algorithmic solutions within traditional computing, or that are unapproachable due to the myriad combinatorial possibilities (Rich and Knight, 1991).

Among the most commonly used algorithms in the aforementioned educational research on academic performance, classification, or early school dropout prediction, we find J48, Logistic Regression, Decision Trees, Random Forests, ANN – Multilayer Perceptron, or Naïve Bayes (NB).

When discussing AI applied to emotions within the educational framework, we encounter initiatives that encompass these dimensions of AI, such as the imitative, identificatory, classificatory, or predictive approaches.

The former focuses on enabling a machine to imitate certain human emotions to facilitate interaction (Zhai and Wibowo, 2023). This emotion-based interaction can be aimed at providing feedback to students (Arguedas et al., 2018), or even at detecting those students who are manipulating online activities and acting accordingly, mimicking different emotions based on the type of student behavior with the activities (Baker et al., 2006).

Classification and identification research focus on AI’s ability to recognize emotions in individuals for various purposes, such as evaluative (Vidanaralage et al., 2022), classificatory (Lian, 2023), or as a preliminary step for emotional regulation in individuals (Wetcho and Na-Songkhla, 2022). These classification and identification-oriented uses have also been employed to measure mental health (Xu et al., 2022), sentiment analysis (Peng et al., 2022), or even to identify warning signs regarding mental health, such as identifying depression in college students (Ding et al., 2020). In all of the aforementioned cases, AI can prove to be a key tool as it enables the automatic identification and classification of emotions, which can assist educators and counselors in their task of managing students’ emotions in classrooms with a high number of students.

Other initiatives combine the imitative aspect with identification, whereby emotions in individuals are identified and the machine imitates others in response to enhance interaction (Gaeta et al., 2023; Han et al., 2023). The interaction of these imitative models with students can be carried out in various ways, such as through empathetic agents that can even be integrated into virtual reality systems (Hernández and Ramírez, 2016). This represents an intersection point with another ICT tool, virtual reality, which has also experienced significant growth in its use within the educational domain in recent years (Roda-Segarra et al., 2022).

The research convergence of AI, emotions, and education has been highly productive in recent years, as will be detailed throughout this investigation, yet we do not know if this extensive scientific production is having any sort of social impact. An analysis of the social repercussions of scientific production within a knowledge area is highly relevant considering the concept of knowledge transfer: universities as key actors in knowledge creation and its transfer to companies, the state, and communities (Arias Pérez and Aristizábal Botero, 2011). Focusing on the third axis of knowledge transfer, namely communities, analyzing social media (understood as communication tools in the hands of communities) allows us to gauge the impact of research results on communities.

An analysis exclusively focused on the scientific impact of research overlooks the aspect of the transfer of scientific output, as the research is being analyzed solely for its influence in the academic field, and not in the social one. Therefore, conducting this type of analysis allows for a broader perspective and the study of scientific production from a comprehensive and global viewpoint, enabling us to assess its degree of transfer beyond the international scientific community.

Additionally, such social media analysis can be conducted using specific Information and Communication Technologies (ICT) tools. ICT tools enable the processing of large volumes of information due to the facilities they offer in capturing data and the cost reduction that has occurred in recent decades regarding storage (Liao et al., 2012). This makes them highly suitable tools for analyzing the vast amounts of information related to the social impact of scientific production.

Based on this premise, the present research aims to conduct an analysis of the social impact of the research field concerning the use of AI for emotions within the educational context, comparing it with the scientific impact of the original scientific production, analyzed through scientometric techniques. The goal is to offer a perspective on scientific impact beyond the exclusively scientific realm, bridging towards the social domain and thereby knowledge transfer.

## 2 Objectives

Based on the foregoing, the general objective of the present research is:

- Analyze the social impact of scientific production on the use of AI for emotions in the educational context.

Derived from this general objective, the specific objectives pursued are:

- Select a sample of scientific production that explores the possibilities of AI for emotions in education.
- Obtain social impact records from various databases and store them in a database designed *ad hoc*.
- Compare the bibliometric data of the original sample with the corresponding social impact records.

## 3 Methodology

To address the objectives outlined in the present research, a sample of articles addressing research on emotions in the classroom through AI techniques was first selected. To obtain the sample, the Preferred Reporting Items for Systematic Reviews and Meta-Analysis (PRISMA) methodology guidelines (Page et al., 2021) were consulted. The PRISMA guidelines stipulate that the resulting sample from the original search must undergo three distinct phases, namely identification, screening, and inclusion. The outcome of this screening through the three stages, as described in subsections 3.1, 3.2, and 3.3, will yield the final sample. These three subsections will address the first specific objective, which aimed to select a sample of scientific literature exploring the possibilities of AI for emotions in education. Once this sample is obtained, a database has been designed to store the bibliographic data and their corresponding social impact data obtained from various platforms (Subsection 3.4). This subsection addresses the second specific objective, which aimed to obtain records of social impact from various databases and store them in a specific database. Through this *ad hoc* designed database for the study, which integrates bibliographic data and social impact data, the analyses described in Section 4 will be conducted.

### 3.1 Identification

The first step in the sample identification stage involved selecting the databases for the search. In this case, Scopus and Web of Science (WOS) were utilized. The research was limited to these two databases because both Scopus and WOS cover a wide range of high-quality scientific journals with a high reputation, recognized for their comprehensiveness and rigor in selecting articles for publication. The search string in title, abstract, or keywords consisted of “artificial intelligence” or its acronym, along with the term “emotion” or its plural form. Additionally, one of the following terms related to the educational context had to appear: education, classroom, students, or teachers. The search was not restricted by any time frame or language. Secondary sources and grey literature have not been investigated in the present research. Thus, the search string in both databases resulted as follows:

*(“artificial intelligence” OR “AI”) AND (emotions OR emotion) AND (education OR classroom OR students OR teachers)*

On December 20, 2023, the search yielded 866 documents in Scopus and 959 in Web of Science (WOS). To identify documents for the subsequent screening phase, inclusion criteria were applied: (1) the document had to belong to thematic areas related to social sciences or computer science, (2) its content had to be accessible as open access, and (3) the document must have a Digital Object Identifier (DOI). The reason for including as an inclusion criterion that the research must have a DOI is that the social impact databases used in this study require the DOI of the document to retrieve information about its social impact.

In the case of Scopus, the thematic areas related to inclusion criterion (1) were “social sciences” and “computer science.” A total of 154 documents not belonging to these categories were discarded, along with 547 others not meeting the open access criterion (2). Thus, the Scopus sample size after applying the inclusion criteria was 165 documents.

Regarding WOS, the thematic areas for inclusion criterion (1) were “social sciences and other topics,” “computer science,” “education,” and “educational research.” 125 documents not belonging to these thematic areas were discarded, along with 506 others not meeting the open access criterion (2). The resulting WOS sample size after applying the inclusion criteria was 328 documents.

The samples from Scopus and WOS were combined into a single sample of 493 documents. Duplicate documents (n = 88) were removed, and one document was excluded due to not having a DOI, according to inclusion criterion (1). Thus, the sample for screening comprised n = 404 documents.

### 3.2 Screening

In this phase, the 404 documents were individually reviewed to filter them according to the exclusion criteria: (1) not focusing on emotions, (2) not utilizing AI technologies, and (3) not occurring in an educational context. Consequently, a total of 161 documents were discarded. The breakdown comprised 77 documents that did not meet criterion (1), 23 that did not fulfill criterion (2), and 61 that were unrelated to criterion (3). The final sample of documents included in the research consisted of n = 243.

### 3.3 Inclusion

**Figure 1:**
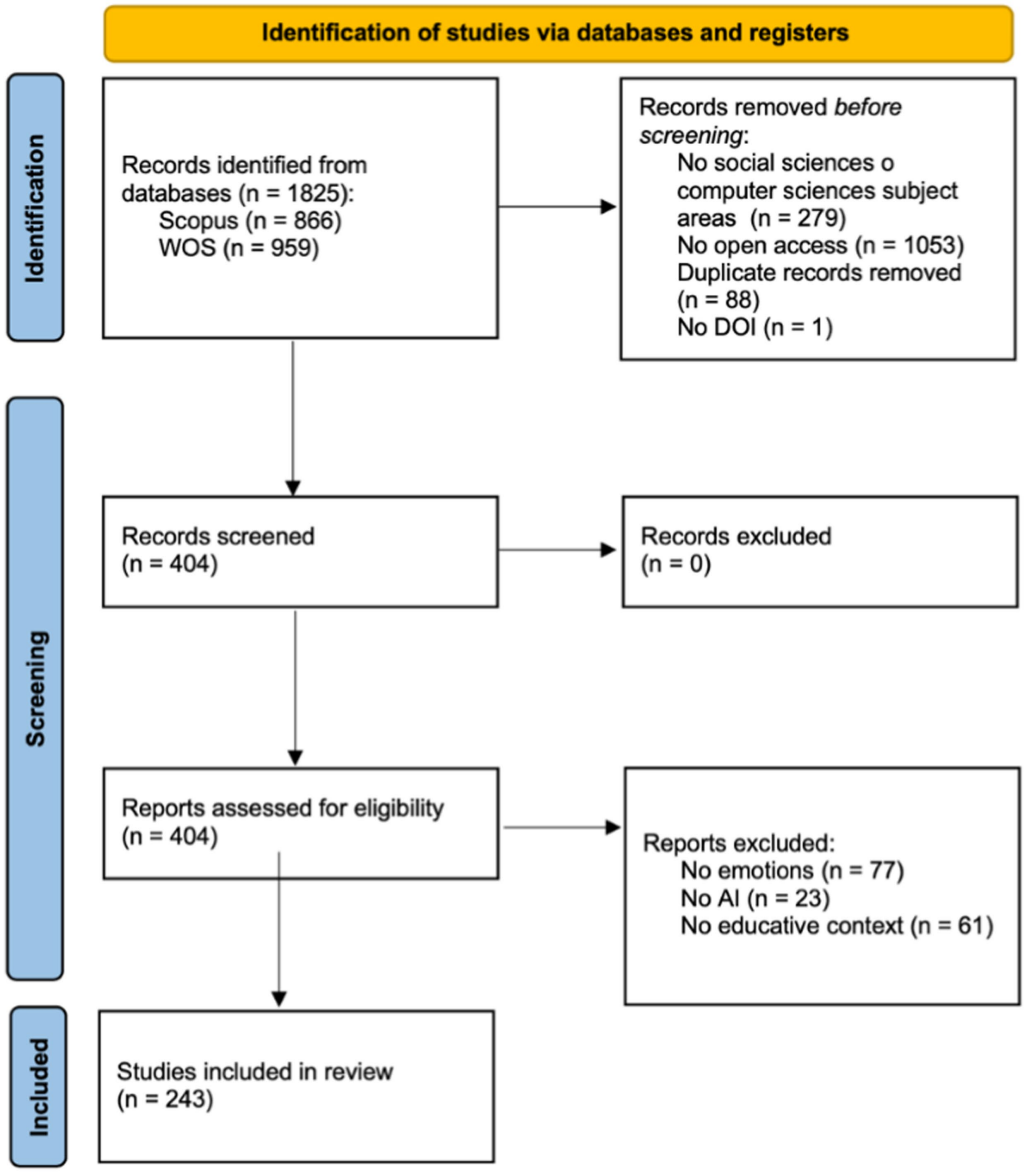
PRISMA flow chart. Source: own production.

The process described leading to the final sample of documents included for the research is summarized in the flowchart (Figure 1), following PRISMA specifications (Page et al., 2021).

The resulting sample of n = 243 documents proceeded to the next phase, in which an *ad hoc* database was designed for the incorporation of the bibliographic data of the sample and could also store the information extracted from different databases that measure social impact.

### 3.4 Design of a database for bibliographic data and corresponding social impact data

The bibliographic data of the resulting sample (title, DOI, abstract, authors and affiliations, publication year, document type, global citations, language, database, and source) were stored in a spreadsheet. However, incorporating data related to social impact required, for flexibility purposes, the design of a relational database that combined both types of data: the bibliographic data and those extracted from the social impact that the scientific production of the sample had received.

**Figure 2:**
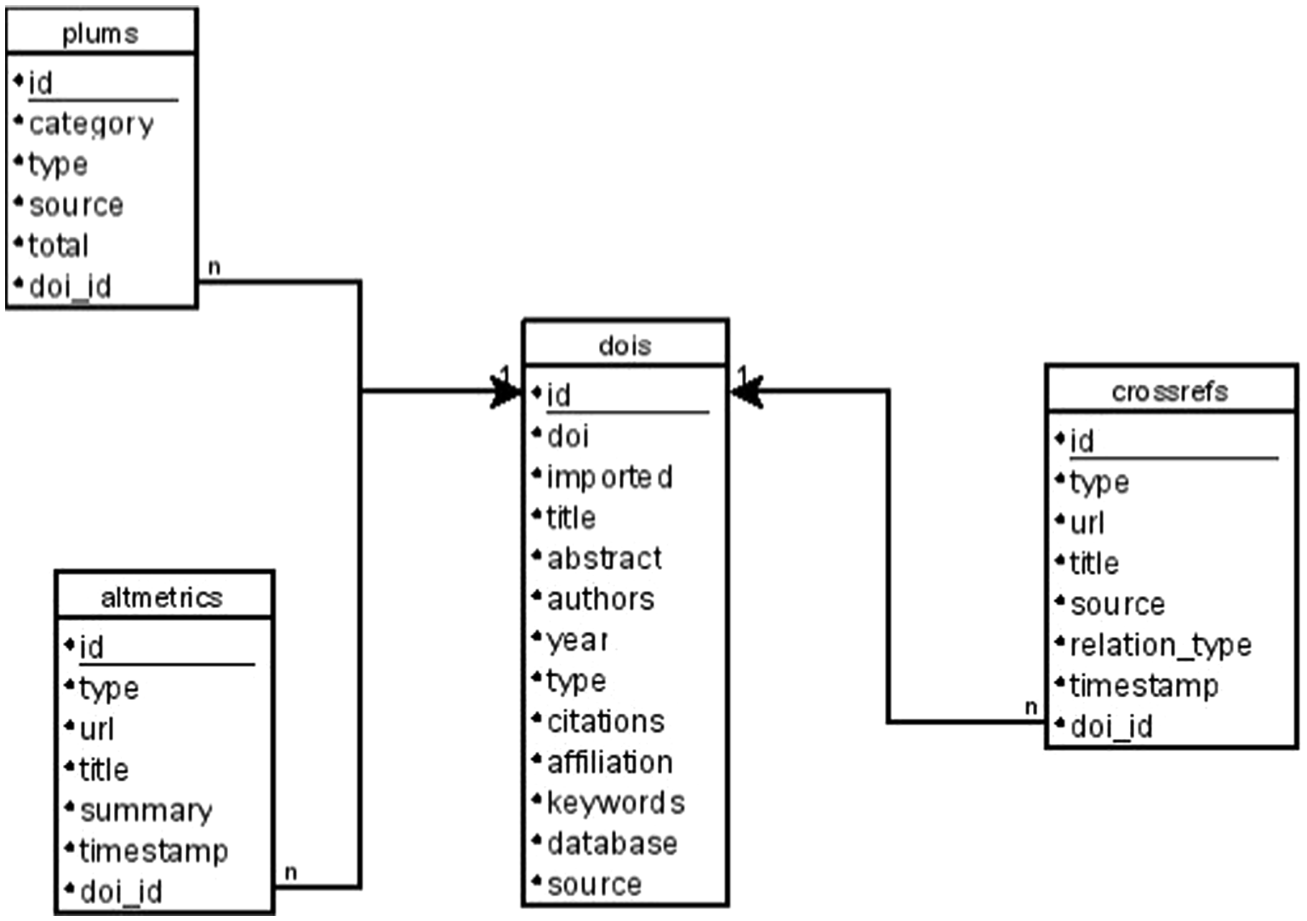
ER model. Source: own production.

As described by Quiroz (2003), a relational database consists of one or more two-dimensional tables, where the columns are the attributes of the data and the rows are each of the records, allowing for the interrelation of attributes from different tables. This relational aspect provides the necessary flexibility to the stated objective of storing bibliographic data in one table, which will be related to the social impact data stored in other separate tables. The composition of the tables, as well as the relationship between their attributes, is captured in an entity-relationship (ER) model, which reflects both the data and their relationships (Cerrada Somolinos et al., 2000). The ER model of the database specifically designed for the present research can be consulted in Figure 2.

In the ER model, it can be identified that the database consists of 4 tables. The first one (*dois*) stores the bibliographic data of the sample obtained in the previous steps. Therefore, it stores information about the title, abstract, authors, publication year, document type (article, review, conference paper, book chapter, book…), citations, affiliations, keywords, the database it comes from (Scopus or WOS), and the source (the journal or book where it was published). Additionally, it includes some necessary fields in a relational database, such as the identifier (*id*), and another field to mark if the social impact data of the document has already been imported (imported, which can have the values true or false).

Regarding the social impact data, three specific databases were utilized: PlumX Metrics,^1^ Altmetric,^2^ and Crossref.^3^

PlumX Metrics, part of Elsevier since 2017, categorizes social impact information about a publication according to 5 categories (Plum, 2024): citations, usage, captures, mentions, and social media. For the present research, the information provided in the usage category and in the captures category was not utilized, as neither provided information regarding social impact, but rather individual usage of research content (for example, the number of times an abstract had been viewed or a document downloaded, the number of times it had been saved as a favorite, etc.). The number of social impact records discarded for this reason was n = 1,508 for usage and n = 10,856 for capture, making a total of 12,364 records discarded due to not having a direct relationship with social impact.

Altmetric, a Digital Science database, classifies social impact information according to the social network where it has occurred. It also records whether there is an impact on policy documents and patents. Specifically, the information it stores regarding scientific publication is as follows (Altmetric, 2024): Facebook, blogs, Google+ (it should be noted that although Google+ is still listed in Altmetric’s information, the Google+ social network was closed in 2019, so social impact records on this network will cover, at most, up to 2019), news, Reddit, Question & Answer forums, Twitter, Youtube, Wikipedia, policies, and patents.

Crossref, on the other hand, was founded in 2000 by various scientific societies and publishers (Crossref, 2024), and its database stores the list of DOIs of those research papers that have referenced the document from which information is sought. Therefore, the information obtainable from Crossref encompasses both the scientific realm and its social impact.

To obtain information from these three databases, Application Programming Interfaces (APIs) provided by the three platforms were utilized. An API exposes services or data through a software application via predefined resources (Stylos et al., 2009), enabling automated access to data and subsequent processing. Both PlumX Metrics and Crossref allowed direct access to their API, but in the case of Altmetric, a request had to be made explaining the intended use of the obtained information.

Therefore, a table was designed in the specific database for each of the social impact databases. In the ER model depicted in Figure 2, we can observe the tables plums, altmetrics, and crossrefs, each with specific attributes based on the information obtained through the APIs.

Regarding the procedure followed for obtaining data from these three databases, it is worth noting that the information they return is not homogeneous, so it cannot be counted in the same manner. In the case of Altmetric and Crossref, one record implies one social impact, but in the case of PlumX, one record implies n social impacts, according to the total field returned by the record. Therefore, in the case of PlumX, the number of records obtained was not counted, but rather the sum of the total fields of each of these records was calculated. This is reflected in the design of the specific table for storing PlumX data, which includes the total field (Figure 2), while the tables for Crossref and Altmetric do not.

Additionally, a common field (doi_id) was included in all three tables, linking the record to the document stored in the dois table. This established a relationship between the social impact data and the document that generated it. This relationship was one-to-many, meaning that a single document could have generated multiple social impact records.

To access the data provided by the three APIs and subsequently store it in the described database, a web application was developed using the PHP language and the Laravel framework. Laravel facilitates application development, adds rigor to development, ensures a coherent architecture, and allows task automation (Laaziri et al., 2019). The procedure followed was as follows: (1) incorporate the DOIs from the spreadsheet into the dois table of the database, (2) the application queried these DOIs one by one and requested the social impact data from the corresponding DOI from the APIs of the three platforms, (3) the results returned by the APIs were stored in the respective tables for PlumX Metrics, Altmetric, and Crossref, (4) and access was made to the Twitter API to obtain information for each of the tweets collected by Altmetric, as this database only provides tweet identifiers without offering information regarding their contents or publication dates. Once these data were stored, data processing continued to obtain the results described in the following section.

## 4 Results

The sample has been analyzed from a comparative perspective, whereby bibliometric aspects of scientific production have been studied concurrently with analyzing social impact aspects most related to these bibliometric aspects. In the bibliometric part of the analysis, not all parameters typically included in a comprehensive bibliometric study have been analyzed, as, for example, following the criteria of Zupic and Čater (2015), since it is not the main objective of the present research, and because not all bibliometric aspects had an equivalent from the social impact analysis. For the bibliometric analysis, the Hecumen tool (Roda-Segarra and Mengual-Andrés, 2023) has been used, allowing a descriptive analysis of the basic aspects of a sample from a bibliometric standpoint.

Thus, in the following sections, the evolution of scientific production is compared with its corresponding social impact (Subsection 4.1), the scientific sources of the sample and their corresponding social sources are compared (Subsection 4.2), the impact of research at both scientific and social levels is examined (Subsection 4.3), and a content analysis is conducted for both scientific and social aspects (Subsection 4.4). These four subsections address the third specific objective, which aimed to compare the bibliometric data of the original sample with the corresponding records of social impact.

### 4.1 Evolution of scientific production in relation to its social impact

For the analysis of the evolution of scientific production and its social impact, a dual descriptive analysis has been employed: firstly, the scientific production has been analyzed exclusively, considering its evolution over time and from the perspective of authors and their relationship with the produced sample. Secondly, the social impact produced by this sample has been analyzed, comparing it temporally with the evolution of its scientific production.

Regarding scientific production exclusively, the first document in the sample dates back to 1993, while the latest is from 2023. Production is very low between 1993 and 2018 (less than 10 articles per year, but in the first 20 years of the sample, only 1 or no articles have been identified per year), but from 2019 onwards, the curve grows exponentially, reaching its peak in 2022 with 81 articles. However, in 2023, it decreases again to 58 documents.

Regarding the types of documents, out of the 243 analyzed, a vast majority (n = 186) were articles, followed by 23 conference papers, 14 proceedings papers, and 10 reviews. In the remaining documents (n = 10), we found early access articles, early access reviews, and only one chapter. The sample was authored by 836 authors who appeared 871 times. Only 26 of the authors produced their entire output solo. Additionally, the production of these 26 authors who published solo amounts to just over one article per author (1.12 articles on average per author who published individually), as only 29 articles were authored by a single person. In contrast, the majority of authors (n = 810) conducted research in combination with others, producing a total of 214 multi-authored articles, with an average of 3.44 authors per article and a collaboration index of 3.79 (authors of multi-authored documents divided by multi-authored documents).

Among these authors, J.M. Harley stands out as the only author in the sample with 6 articles (2.47% of the sample’s production), followed by R. Azevedo, who contributed 4 articles to the sample (1.65%). The rest of the authors maintained 2 works or fewer. These two highest-producing authors (J.M. Harley and R. Azevedo) were the most collaborative in the sample: all four articles by R. Azevedo were also co-authored by J.M. Harley. However, their dominance indices (articles with another author while being the first author / articles with another author) stand at 0.67 for J.M. Harley and 0 for R. Azevedo (implying that R. Azevedo did not co-author any multi-authored documents while being the first author).

**Figure 3:**
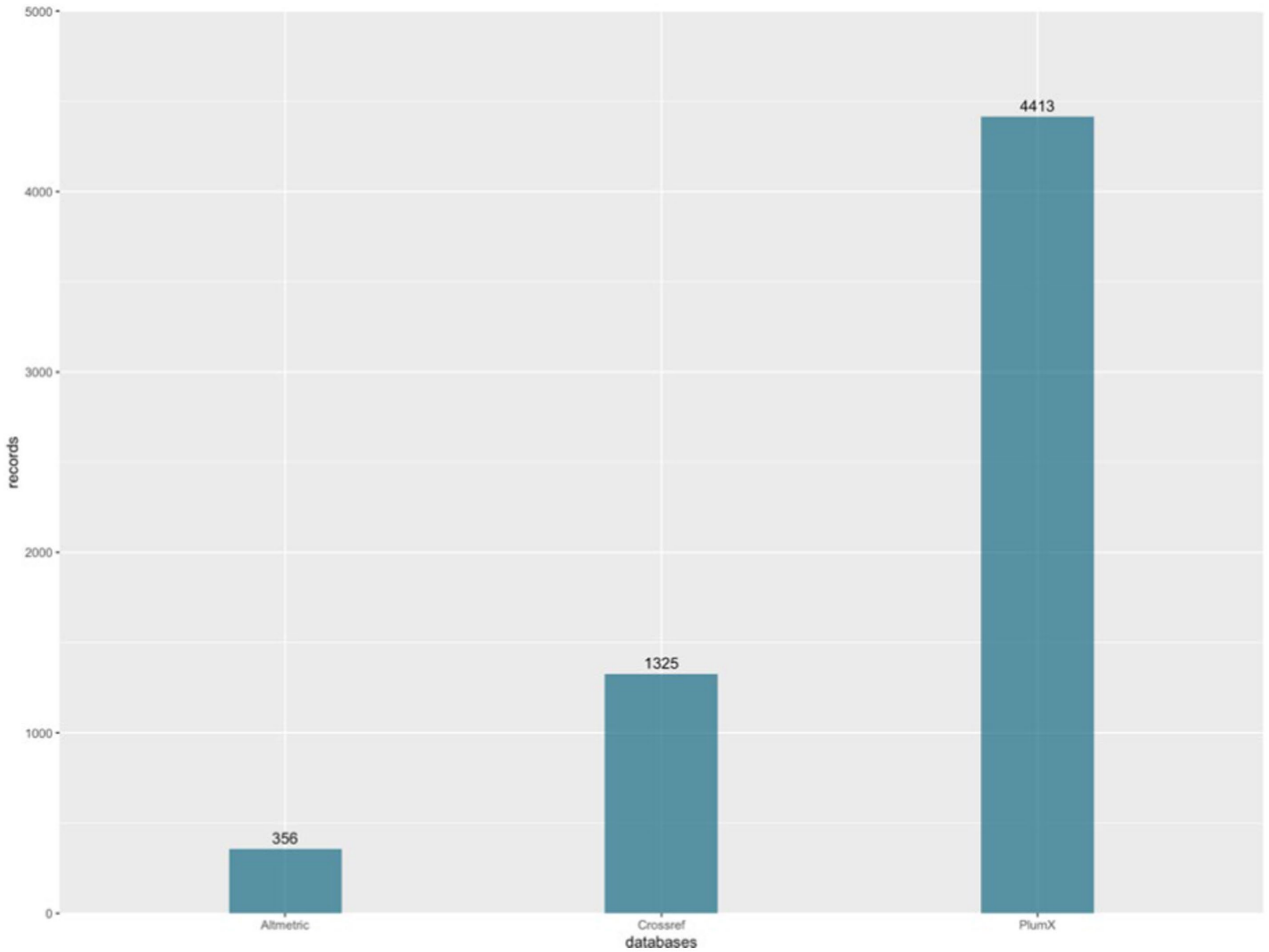
Social impact records by database. Source: own production.

Regarding geographical aspects, 32.51% of the documents were primarily authored by individuals affiliated with institutions in China, followed by 7.41% of the sample with authors affiliated with the United States, and the same percentage of documents with first authors affiliated with Spain. The authors predominantly wrote their research in English (n = 240), with n = 1 each for Russian, Spanish, and Ukrainian. This scientific output generated a total of 6,094 social impact records, considering the records stored in the three databases used (Altmetric, Crossref, and PlumX). This yields an average of 25.08 social impact records per document analyzed from the sample. The contribution of each of the three databases used to the sample of 6,094 social impact records can be visualized graphically in Figure 3, corresponding to the social impact records obtained from PlumX (n = 4,413), followed by those obtained from Crossref (n = 1,325), and lastly, Altmetric (n = 356).

On the other hand, social impact records that have temporal registration, such as Altmetric and Crossref (since PlumX does not provide information on the dates of the records it stores), begin in 2016 (n = 5) and grow with a curve similar to that of production, although with a much steeper slope. In fact, the highest number of social impact records is found in 2022, with 740 records, more than 9 times higher than the scientific production of the same year. Its decline in 2023 is similar to the decline in scientific production, with 625 records.

**Figure 4:**
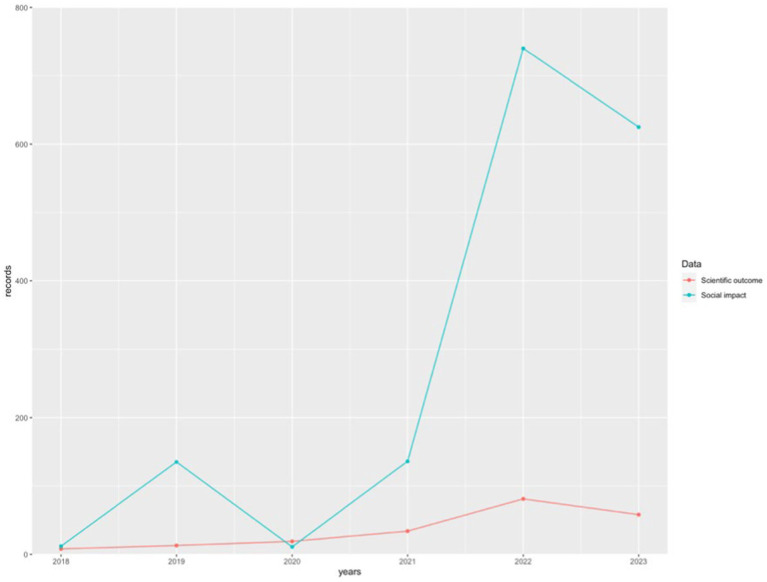
Comparative between scientific output and social impact. Source: own production.

The visual comparison between the evolution of scientific production and the evolution of social impact can be observed in Figure 4.

### 4.2 Scientific and social sources

For the analysis of sources, firstly, a list of the sources that have published the most research from the sample was compiled. Subsequently, a detailed analysis of the corresponding social impact records was carried out, describing the different sources that contributed to the records based on the database from which they were obtained.

The source with the highest number of research publications in the sample was *IEEE Access*, with a total of 19 documents, accounting for 7.82% of the total sample. In second place, we find *Lecture Notes in Computer Science* with 14 documents (5.76%), followed by *Scientific Programming*, with 10 publications, representing 4.12% of the sample. These three sources, along with the remaining 7 sources where the highest number of research publications from the sample were published, can be analyzed in Table 1, which accounts for 34.58% of the total sample.

**Table 1:** Sources of scientific production.

Source	Documents	%
IEEE Access	19	7.82%
Lecture Notes in Computer Science	14	5.76%
Scientific Programming	10	4.12%
Mobile Information Systems	8	3.29%
Sensors	8	3.29%
Applied Sciences-Basel	6	2.47%
Multimedia Tools and Applications	6	2.47%
Journal of Environmental and Public Health	5	2.06%
Computational Intelligence and Neuroscience	4	1.65%
International Journal of Artificial Intelligence in Education	4	1.65%

Source: own production.

**Figure 5:**
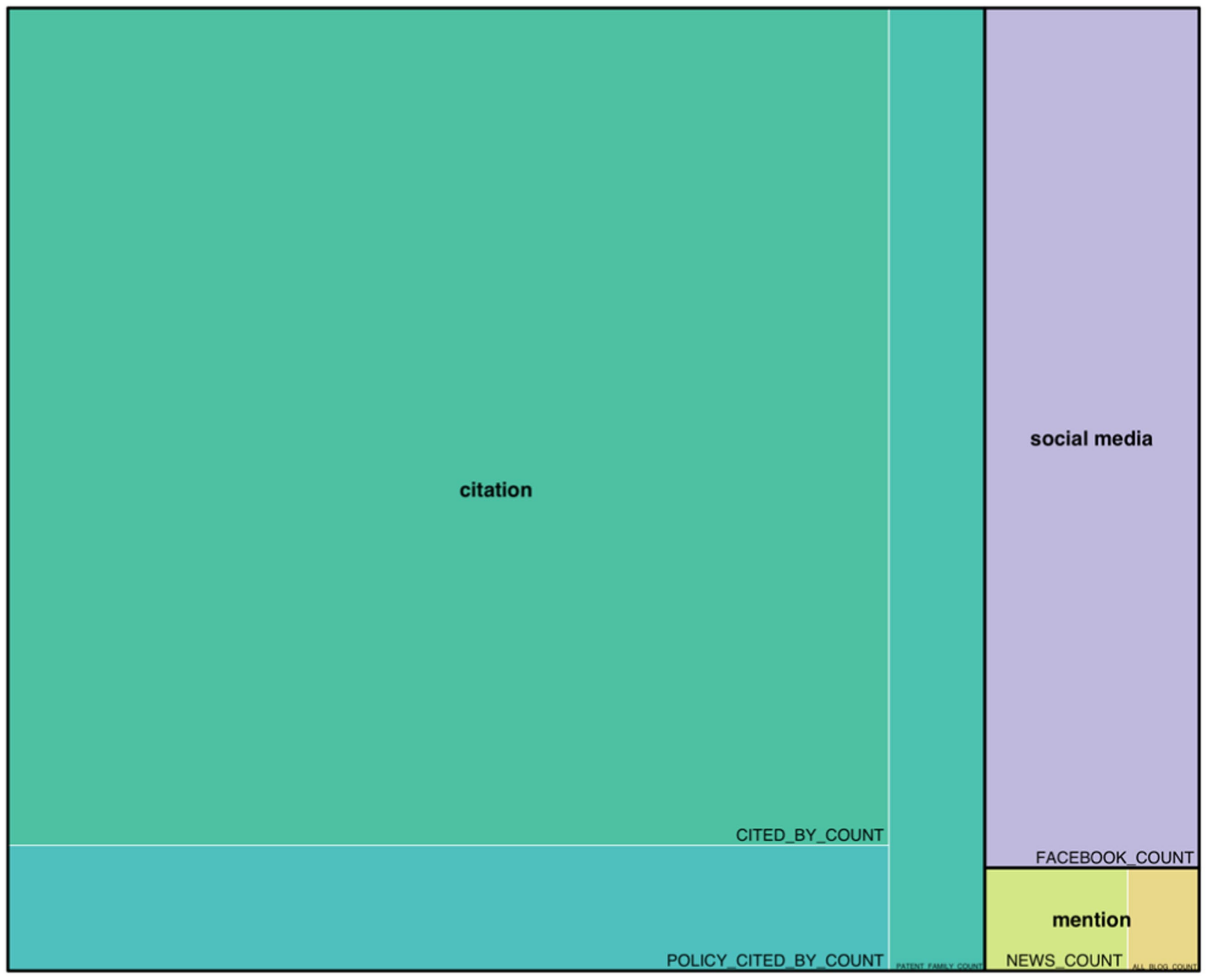
PlumX records by category. Source: own production.

Regarding the analysis of social impact sources, due to the heterogeneity of the information provided by the three databases used, an individualized analysis of social impact records has been conducted. Firstly, Figure 5 reflects the percentage of each social impact category as stored by PlumX. The category with the highest number of social impact records is “citation” (*n* = 3,769), with the highest number of records corresponding to citations in scientific databases such as Scopus or SciELO (n = 3,745), to a lesser extent, citations in policy documents (n = 23), and only one reference in a single patent. The last two categories are “social media” (n = 558) and “mention” (*n* = 86). All “social media” records correspond to posts on Facebook, while in the case of “mention,” the records obtained in PlumX are broken down into news mentions (*n* = 71) and blog posts (*n* = 15).

Regarding the records obtained from Crossref, almost all of them correspond to citations in other scientific publications (*n* = 1,274), followed by a much lower number of records from Datacite (*n* = 43), and lastly, from news sources (*n* = 2) or Wikipedia (*n* = 1).

**Figure 6:**
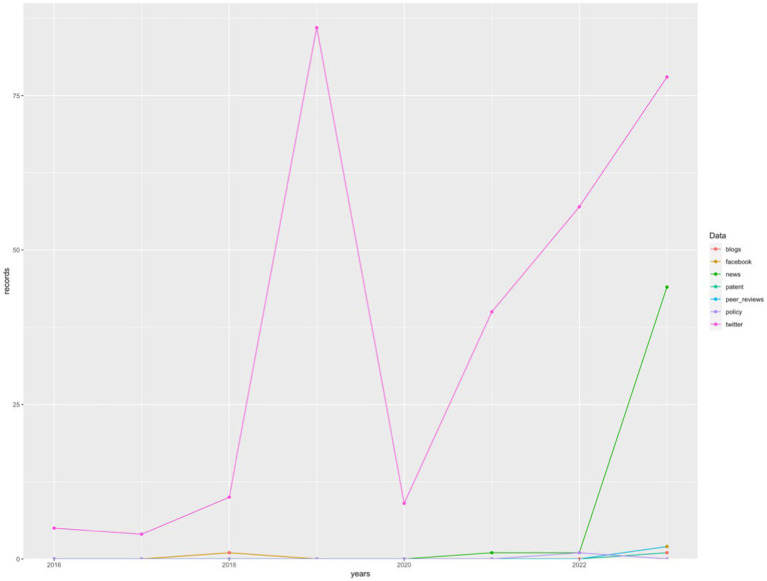
Number of records by category in Altmetric. Source: own production.

The last database analyzed, Altmetric, despite contributing the fewest social impact records to the sample, provides quite a bit of information about each record. In addition to the category to which the record belongs (Facebook, blogs, Google+, news, Reddit, Q&A forums, Twitter, YouTube, Wikipedia, policies, or patents), it also provides information about the title and abstract of the original record, as well as the date it was published. Based on this last piece of data, the chronological evolution of the number of records in each category has been analyzed and graphically represented in Figure 6, where the significant increase in the “news” category during the year 2023 can be observed and a large majority of social records on Twitter with a higher peak in 2019 (*n* = 86).

Regarding the sources of news that have published the most information on the scientific production of the sample, only two have published more than one piece of news. These sources are *News Azi* (*n* = 2) and *Phys.org* (*n* = 2). *Phys.org* stands out as part of the *Science X* network of websites specialized in scientific dissemination, with 10 million monthly readers and around 200 daily articles (Science X, 2024). Additionally, a high number of repeated news items across various sources have been identified. “Robots are everywhere – improving how they communicate with people could advance human-robot collaboration” was the most copied news item, appearing in 33 different sources (*Arizona Daily Star, Beatrice Daily Sun, Billings Gazette, Bozeman Daily Chronicle, Columbus Telegram, Daily Journal, Dispatch-Argus, EconoTimes, Gazette-Times, GCN, Houston Chronicle, Idaho Press, Independent Record, Lincoln Journal Star, Missoulian, New Canaan Advertiser, News Azi, Northwest Indiana Times, Rapid City Journal, San Antonio Express-News, Seattle Post-Intelligencer, SFGate, Shelton Herald, St. Louis Post-Dispatch*, *tdn.com**, The Bismarck Tribune, The Buffalo News, The Conversation, The Darien Times, The Preston Citizen, The Southern Illinoisan, WFMZ-TV 69*, and *Yahoo! News*). “Technology with empathy: using conversational agents in education” appears in 4 sources (*AlphaGalileo, EurekAlert!, MSN*, and *Phys.org*). Lastly, “Improving how robots communicate with people” appears duplicated in 2 sources (*Space Daily* and *Terra Daily*).

### 4.3 Impact of research at the scientific and social levels

For the study of the impact of production, a dual analysis has been conducted: firstly, the impact in the scientific domain has been examined, using scientometric criteria to measure the impact of a publication based on the number of citations it has received; based on this study, a list of the 10 publications with the highest scientific impact has been compiled. Secondly, the same study has been conducted, but from the perspective of social impact. For this, the number of social records obtained by each publication has been considered instead of the number of citations. Similarly to the first case, another list has been prepared with the 10 publications that have had the greatest social impact. Subsequently, both lists have been compared to establish points of similarity.

Regarding the first analysis of scientific impact based on the most globally cited documents in the sample, the top three works with over 100 citations are “Engagement detection in online learning: a review” (Dewan et al., 2019) with 122 citations, “Towards Emotionally Aware AI Smart Classroom: Current Issues and Directions for Engineering and Education” (Kim et al., 2018) with 110 citations, and “Adapting to when students game an intelligent tutoring system” (Baker et al., 2006) with 103 citations. The list of the top 10 most cited documents can be observed in Table 2.

**Table 2:** Top 10 most cited documents.

Title	Global citations	Global citations per year	Authors
Engagement detection in online learning: a review	122	3.94	Dewan et al. (2019)
Towards Emotionally Aware AI Smart Classroom: Current Issues and Directions for Engineering and Education	110	3.55	Kim et al. (2018)
Adapting to when students game an intelligent tutoring system	103	3.32	Baker et al. (2006)
Conversations with AutoTutor Help Students Learn	95	3.06	Graesser (2016)
Detecting and Addressing Frustration in a Serious Game for Military Training	65	2.1	DeFalco et al. (2018)
LSTM-Based Emotion Detection Using Physiological Signals: IoT Framework for Healthcare and Distance Learning in COVID-19	62	2	Awais et al. (2020)
Artificial intelligence in early childhood education: A scoping review	60	1.94	Su and Yang (2022)
Sentiment analysis in MOOCs: A case study	57	1.84	Moreno-Marcos et al. (2018)
Modeling students’ emotions from cognitive appraisal in educational games	54	1.74	Conati and Zhou (2002)
Developing Emotion-Aware, Advanced Learning Technologies: A Taxonomy of Approaches and Features	52	1.68	Harley et al. (2017)

Source: own production.

For the second analysis, the number of social impact records from the three databases used has been considered. Based on these data, the document with the highest social impact has been “Emotion-Based Adaptive Learning Systems” (Taurah et al., 2020) with 326 social impact references, accounting for 6.50% of the total social impact references. This research does not appear in the list of the top 10 most cited documents in the scientific domain. The next work is “Adapting to when students game an intelligent tutoring system” (Baker et al., 2006), with 276 references (5.51%), which also coincides with the third most cited work in the scientific domain. The third document is “Conversations with AutoTutor Help Students Learn” (Graesser, 2016) with 233 references and 4.65%. In this case, we find the work at the top of the list of the most cited research in the scientific domain. The complete list of the top 10 publications with the most social impact references in the PlumX, Altmetric, and Crossref databases can be found in Table 3.

**Table 3:** Top 10 documents with social impact.

Title	References	%	Authors
Emotion-Based Adaptive Learning Systems	326	6.50	Taurah et al. (2020)
Adapting to when students game an intelligent tutoring system	276	5.51	Baker et al. (2006)
Conversations with AutoTutor Help Students Learn	233	4.65	Graesser (2016)
Towards Emotionally Aware AI Smart Classroom: Current Issues and Directions for Engineering and Education	228	4.55	Kim et al. (2018)
Humanoid Robots as Teachers and a Proposed Code of Practice	181	3.61	Newton and Newton (2019)
Emotional AI and EdTech: serving the public good?	177	3.53	McStay (2020)
Predicting affect from gaze data during interaction with an intelligent tutoring system	177	3.53	Jaques et al. (2014)
Predicting regulatory activities for socially shared regulation to optimize collaborative learning	159	3.17	Järvelä et al. (2023)
Building pipelines for educational data using AI and multimodal analytics: A grey-box approach	156	3.11	Sharma et al. (2019)
Towards AI-powered personalization in MOOC learning	153	3.05	Yu et al. (2017)

Source: own production.

**Figure 7:**
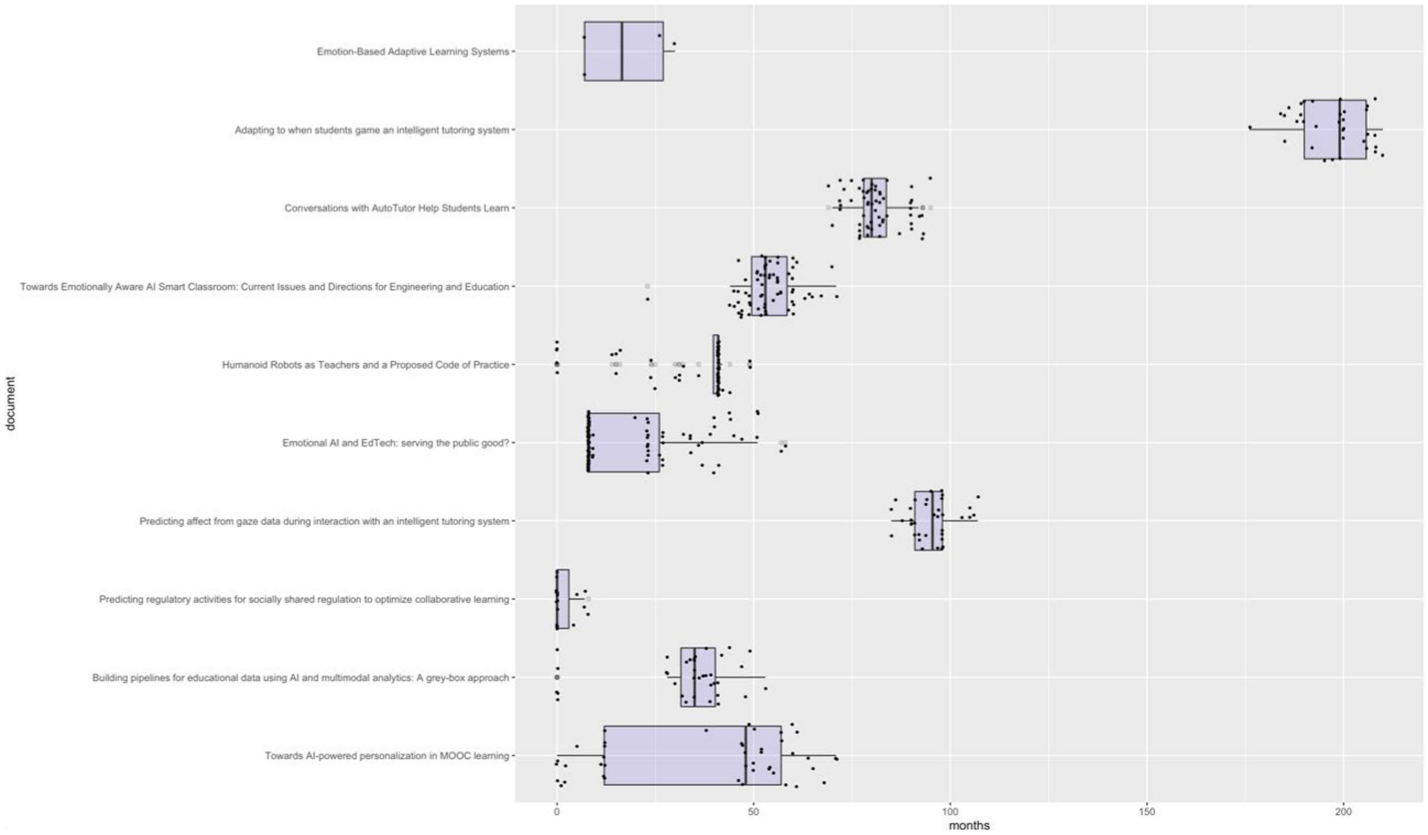
Analysis of the publication lag of social records in relation to research publication. Source: own production.

However, the social impact of the 10 most scientifically impactful documents has not been immediate. To analyze this issue, the lag in months between the date of original publication and the dates of social records from Altmetric and Crossref has been calculated (as PlumX does not provide the publication date of the record). To do this, the boxplot in Figure 7 has been developed, where the lag in months of the first social record, the last one, the first quartile, the third quartile, and the median are represented.

Comparing the results from Tables 2, 3, we find that only 3 of the top 10 most cited publications in the scientific domain coincide with the top 10 publications that have had the most social impact. These 3 publications, which have had both scientific relevance and social impact simultaneously, are “Towards Emotionally Aware AI Smart Classroom: Current Issues and Directions for Engineering and Education” (Kim et al., 2018), “Adapting to when students game an intelligent tutoring system” (Baker et al., 2006), and “Conversations with AutoTutor Help Students Learn” (Graesser, 2016).

If we break down the social impact of studies based on their social source, the 10 research papers that had the greatest impact on Twitter (tweets or retweets) were “Emotional AI and EdTech: serving the public good?” (McStay, 2020) with 86 records, “Deploying a robotic positive psychology coach to improve college students’ psychological well-being” with 22 records (Jeong et al., 2023), “Sentiment analysis for formative assessment in higher education: a systematic literature review” with 14 records (Grimalt-Álvaro and Usart, 2023), “Towards AI-powered personalization in MOOC learning” with 14 records (Yu et al., 2017), “Predicting regulatory activities for socially shared regulation to optimize collaborative learning” with 13 records (Järvelä et al., 2023), “Engagement detection in online learning: a review” with 12 records (Dewan et al., 2019), “Artificial intelligence in early childhood education: A scoping review” with 12 records (Su and Yang, 2022), “Humanoid Robots as Teachers and a Proposed Code of Practice” with 9 records (Newton and Newton, 2019), “Sentiment Analysis of Students’ Feedback with NLP and Deep Learning: A Systematic Mapping Study” with 8 records (Kastrati et al., 2021), and “Deep Learning-Based Cost-Effective and Responsive Robot for Autism Treatment” with 8 records (Singh et al., 2023).

From the list of works with the most impact on Twitter, only the studies by Dewan et al. (2019) and Su and Yang (2022) appear in the list of the top 10 most cited articles scientifically, contrasting their scientific impacts (*n* = 122, *n* = 60 respectively) with their impacts on Twitter (*n* = 12, *n* = 12 respectively).

Table 4 presents an analysis of the top 10 most retweeted social contents, that is, those with the highest impact within the same social network. This table reflects the referenced research, the content of the original tweet (simplified), the Twitter user, and the number of retweets.

**Table 4:** Top 10 tweets more retweeted.

Document	Tweet	Retweets	Twitter user
*Emotional AI and EdTech: serving the public good?* (McStay, 2020)	New paper alert: If you have an interest in emotional AI, datafication, child rights and/or edtech, you might enjoy this	22	Andrew McStay
*Emotional AI and EdTech: serving the public good?* (McStay, 2020)	Emotion AI & facial recognition in education - two papers just out	16	Ben Williamson
*Emotional AI and EdTech: serving the public good?* (McStay, 2020)	Fortunately no one has aims to use facial recognition-based emotion AI to assess kids in education … oh wait	14	Ben Williamson
*Predicting regulatory activities for socially shared regulation to optimize collaborative learning* (Järvelä et al., 2023)	We used AI-based methods to predict regulatory activities and found patterns of socially shared regulation in collaborative learning	11	Sanna Järvelä
*Sentiment analysis for formative assessment in higher education: a systematic literature review* (Grimalt-Álvaro and Usart, 2023)	Voleu saber com es fa servir el Sentiment Analisis en educació superior?	8	Mireia Usart
*Emotional AI and EdTech: serving the public good?* (McStay, 2020)	A really good examination of emotion AI in education, its 20+ year history, issues of privacy, child rights and other ethical and legal concerns, plus its basis in highly contestable psychology of basic emotions and facial coding	8	Ben Williamson
*Deploying a robotic positive psychology coach to improve college students’ psychological well-being* (Jeong et al., 2023)	#Paper Deploying a #robotic positive psychology coach to improve college students’ #psychological well-being.	7	eHealth Center UOC
*Engagement detection in online learning: a review* (Dewan et al., 2019)	Free article! Engagement detection in online learning: a review	7	Springer Education
*Building pipelines for educational data using AI and multimodal analytics: A “grey-box” approach* (Sharma et al., 2019)	Our #BJET paper is just published and is available in OA, it proposes a methodology for building machine learning pipelines for multimodal learning analytics.	5	Learner-Computer Interaction Lab
*Technology Enhanced Learning Using Humanoid Robots* (Reforgiato Recupero, 2021)	A new paper published by Diego Reforgiato Recupero from Italy.	5	Future Internet

Source: own production.

Regarding the studies that had the most social impact in terms of news, we cannot compile a list of the top 10, as only 4 works were echoed in news sources. These were “Humanoid Robots as Teachers and a Proposed Code of Practice” with 44 news articles (Newton and Newton, 2019), “Empathic pedagogical conversational agents: A systematic literature review” with 5 news articles (Ortega-Ochoa et al., 2023), “Artificial Intelligence in education: Using heart rate variability (HRV) as a biomarker to assess emotions objectively” with 1 news article (Chung et al., 2021), and “Prediction of Academic Performance of Students in Online Live Classroom Interactions - An Analysis Using Natural Language Processing and Deep Learning Methods” with 1 news article (Zhen et al., 2023).

### 4.4 Analysis of scientific and social content

The scientific production and the records of social impact have been analyzed in terms of their content. To achieve this, the most frequently occurring terms have been extracted from the titles and abstracts of the scientific production. Subsequently, the same process has been applied to the contents of the social impact records, focusing exclusively on those from Altmetric, as it is the only one of the three databases used that provides such data. Lists of the most commonly used words, both in scientific and social contexts, have been compiled, and their temporal evolution over the last 10 years has been represented.

**Figure 8:**
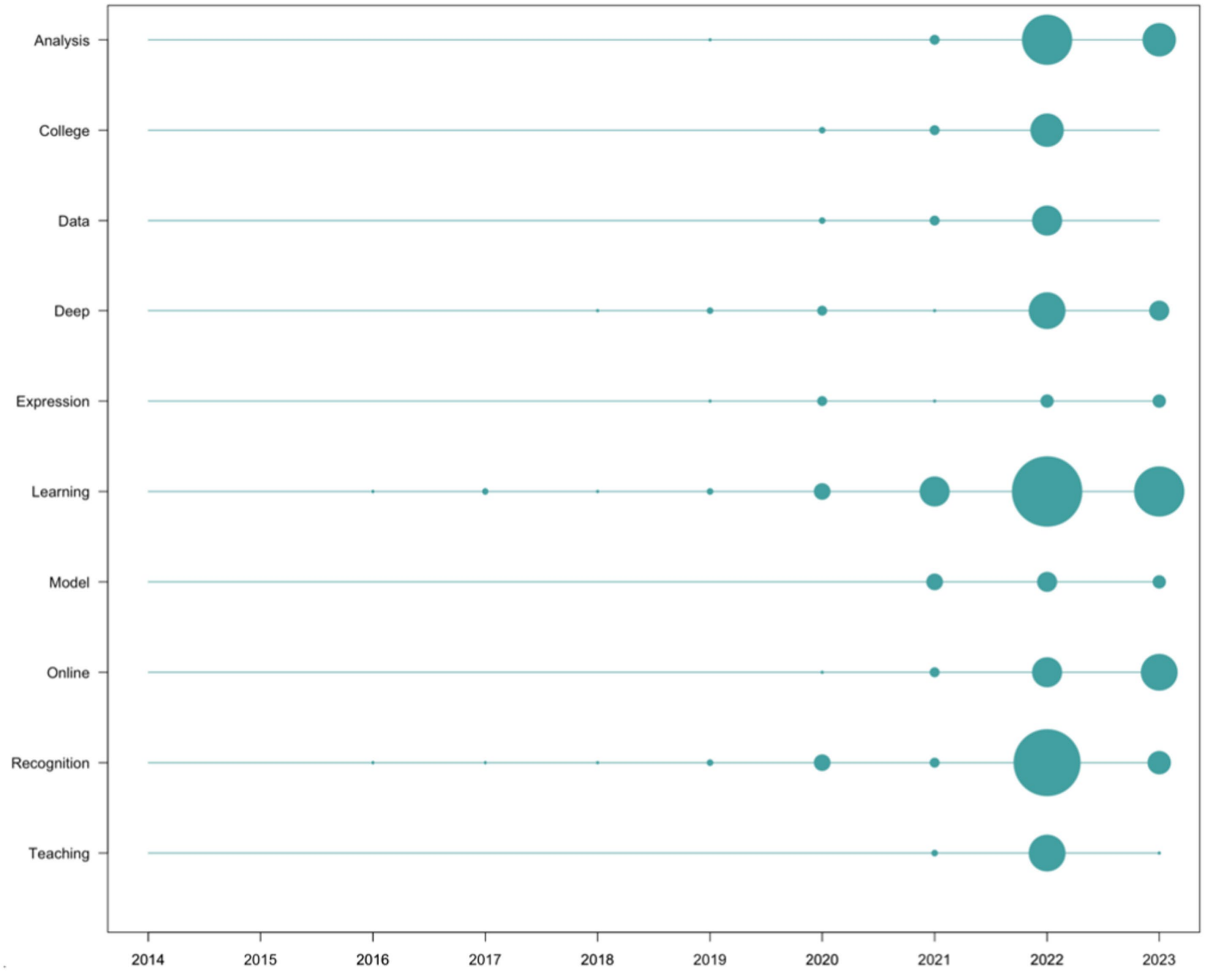
Scientific research word frequency per year. Source: own production.

Regarding the terms that appeared most frequently in the scientific production, in descending order of appearance frequency, we find “Learning” (*n* = 85), “Analysis” (*n* = 43), “Recognition” (*n* = 40), “Deep” (*n* = 24), “Online” (*n* = 24), “College” (*n* = 15), “Model” (*n* = 15), “Data” (*n* = 14), “Teaching” (*n* = 14), and “Expression” (*n* = 13). From this list, content-empty words (connectors, conjunctions, etc.) have been removed, along with those directly related to search strings (artificial intelligence, education, and emotion, as well as their variants). Their temporal evolution has been depicted in Figure 8.

**Figure 9:**
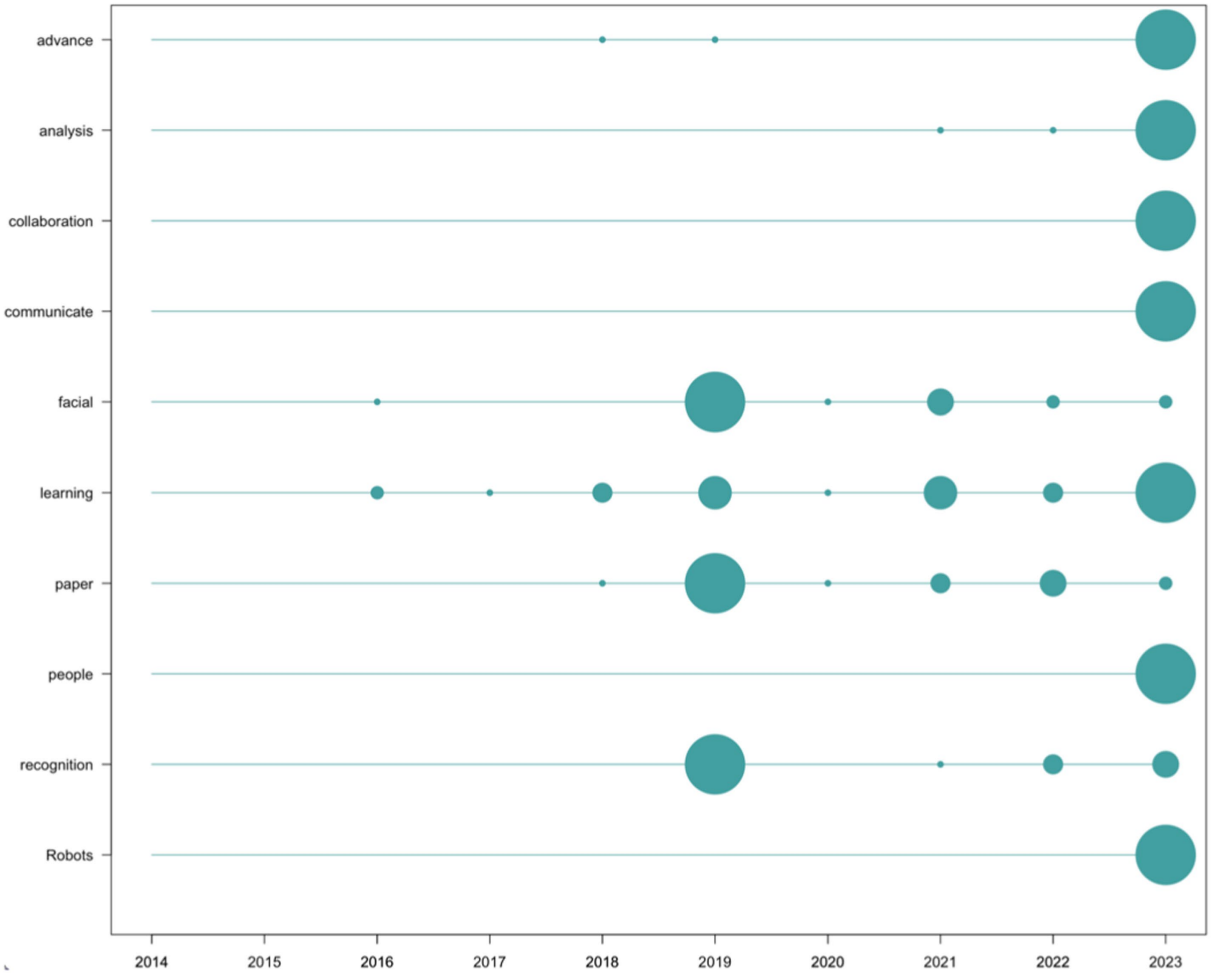
Frequency of words in social impact registers per year. Source: own production.

The terms most frequently used in the records of social impact are “Robots” (*n* = 86), “facial” (*n* = 62), “learning” (*n* = 53), “analysis” (*n* = 47), “recognition” (*n* = 45), “paper” (*n* = 44), “advance” (*n* = 43), “communicate” (*n* = 43), “people” (*n* = 43), and “collaboration” (*n* = 42). This list has been filtered in the same way as the list of words from scientific production (removing content-empty words as well as those directly related to the search). It has been graphically represented in Figure 9.

## 5 Discussion

First, we must highlight the high rate of social impact records in relation to the number of publications, which averages at 25.08 social impact records per analyzed document. The turning point in the scientific production of AI and emotions in education is significant, beginning in 2019 when production starts to increase significantly, with a percentage increase of 62.50% compared to 2018. The peak in scientific production will be reached in 2022, with a 138.24% increase compared to 2021. Focusing on social impact, the increase in social interest shifts two years later than scientific production, starting in 2021 and reaching its peak in 2022 (as seen in scientific production), with a huge increase of 604% compared to the previous year. One reason for the sudden interest in research on AI applied to emotions in education can be found in the psychological effects of the COVID-19 pandemic on the population. In this regard, Pedrosa et al. (2020) state that both the effects of measures taken to control the spread of the COVID-19 pandemic and the implications of the disease itself led to emotional disorders such as fear, anxiety, depression, or suicidal ideation. Additionally, educational institutions had to transition from in-person teaching to remote learning using ICT in a short period of time (Day et al., 2021), making it difficult to address students’ emotional problems in person. Under these conditions, some initiatives of AI applied to managing students’ emotions may have emerged as a solution to the problems arising from the COVID-19 pandemic.

This would align with the fact that in 2023, interest in this field begins to decline, as we find a percentage reduction of 28.40%. The same occurs regarding its social impact, which decreased by 15.54% compared to the previous year. This reduction can hardly be attributed to the timing of the research, as the sample was obtained on December 20, 2023, covering practically the entire year of 2023.

The majority of scientific production (76.54%) consists of articles, far ahead of conference papers at 9.47%. It is noteworthy that 0.41% of the analyzed material comprises chapters, with this percentage corresponding to the sole chapter in the sample.

Similarly, 96.89% of authors conducted research collaboratively, indicating a certain degree of complexity in such initiatives, which demand collaboration among diverse professionals. This may reflect the interdisciplinary nature of projects related to AI and emotions, necessitating the involvement of educators, computer scientists, psychologists, and others. Higher collaboration rates have been observed in other bibliometric analyses concerning the implementation of complex technologies in classrooms, such as virtual reality for educational purposes (Roda-Segarra et al., 2022).

China dominates in terms of publication numbers in this field, with researchers from China accounting for 32.51% of the sample. The percentage significantly drops to 7.41% for both the United States and Spain. The high proportion of research originating from China is notable, particularly in comparison to the next country in terms of affiliations, aligning with findings from other studies related to AI and education. For instance, Roda-Segarra et al. (2024) identified 20% of authors affiliated with institutions in China in a study examining the use of AI for predicting school dropout rates.

However, this dominance in terms of country affiliation of the sample does not reflect in the language used in publication, as 98.77% of the articles were written in English and only 3 articles were written in another language, none of which were Chinese (the three languages different from English were Russian, Spanish, and Ukrainian). Thus, based on these first author affiliation country figures, it can be inferred that there is significant interest in the field of AI applied to education in China, although when it comes to publishing, they prioritize the international dissemination of their research by opting for English.

Regarding the small percentage of social impact records obtained through the Altmetric database (5.84%), compared to PlumX and Crossref (whose sum constitutes 94.16% of the social impact records), it is worth mentioning that both PlumX and Crossref also include citation records within the scientific realm, not just those of social impact.

The most significant aspect of the journals where the scientific publications have appeared is that the top 10 journals that have published the most account for 34.58% of the total sample of publications. This list is led by *IEEE Access*. In fact, out of the total of 144 different sources in the sample, a large percentage of them (*n* = 114, accounting for 79.17%) only published one document, a fact that could be an indicator of the low specialization of scientific publications in the field of research on the use of AI for emotions in the classroom. The novelty of the field of study, the effect of a passing trend or interest, or the temporary concern about the psychological effects of the COVID-19 pandemic mentioned earlier could result in the highlighted data, such as a high number of sources that only publish a single article.

One reason behind this significant social impact when saving publications to online services can be found in the significant news coverage of the field of AI applied to emotions in education. This assertion is supported by data obtained through Altmetric, reflecting a 4,300% increase in the number of news related to the field of study in 2023 (Figure 6). In the same Figure 6, it stands out that Twitter has been the largest source of social impact records, with a peak in 2019 (*n* = 86) and a rising trend from 2020 (*n* = 9) to 2023 (*n* = 78), showing an increase during this period of 766.67%.

As described in Section 3.4, it is important to note that Altmetric does not provide a list of sources from which it obtains publications, so the data should be interpreted with caution as it may vary depending on the news services Altmetric relies on. It should also be noted that the majority of news sources only published one news item about the sample (only *News Anzi* and *Phys.org* published two news items each), reflecting a low degree of specialization of news sources regarding the analyzed topic. Indeed, only the work by Newton and Newton (2019) appeared in the list of the most referenced research on Twitter, and none of the works echoed in the news appeared among the top 10 works with the most scientific impact.

The analyzed data also revealed a high degree of news articles that entirely copied their content from other news sources, likely utilizing some form of automatic content aggregation system. The news article “Robots are everywhere – improving how they communicate with people could advance human-robot collaboration” appeared with the same content in 33 different sources, followed by “Technology with empathy: using conversational agents in education,” which appeared copied in 4 sources, and “Improving how robots communicate with people,” which appeared in 2 different sources. Therefore, 76.47% of the news that echoed the scientific sample were not original, but were copied from other news sources.

Regarding the analysis of impact, both scientific and social, it is noteworthy that only 33.33% of the top 10 most cited documents in the scientific field coincide with the 10 documents with the highest social impact. This implies that in the list of the top 10 documents with the most significant scientific impact from a scientometric perspective, only 1 in every 3 had the corresponding social impact, indicating a lack of alignment between the criteria that grant a publication high scientific impact and significant social repercussion in the field of study. This is reinforced by the data analyzed at the content level, where none of the most commonly used words in scientific research (Figure 8) appears in the list of the most commonly used words in social impact records (Figure 9). While the most frequently occurring words in scientific publications revolve around more technical concepts (for example, “Deep Learning,” “Model, Data,” or “Recognition”), those in social impact records are more media-oriented (“advance,” “collaboration,” “communicate,” “people,” “recognition”). The appearance of the term “robots,” while it does not appear at all in the scientific literature, hinting at the perception that may exist in popular culture regarding the concept of AI.

The analysis conducted also shows that there is significant variability in the lag in months for scientific research to make an impact on social media. According to the analysis of the 10 most scientifically impactful documents, we find research whose first social impact occurred in the same month of publication (Yu et al., 2017; Newton and Newton, 2019; Sharma et al., 2019; Järvelä et al., 2023), while in others, the first social impact occurred more than a year after publication (Jaques et al., 2014; Graesser, 2016; Kim et al., 2018). Notably, the case of the research “Predicting affect from gaze data during interaction with an intelligent tutoring system” (Jaques et al., 2014), whose first social impact was 7 years and 1 month after its publication, with a 25th percentile of 91 months, a 75th percentile of 98 months, a median of 95.5 months, and a last record of social impact 8 years and 11 months after its publication. Other publications show much more time-concentrated social impact records, such as the work of Newton and Newton (2019), with a 25th percentile of 39.75, and both a 75th percentile and median of 41, and the research by Graesser (2016), with a 25th percentile of 78, a 75th percentile of 83.75, and a median of 80.

Regarding the data obtained after analyzing the impact within a single social network like Twitter, a lack of alignment between the scientific impact of the research and its social impact is observed again: 3 of the 10 most retweeted records were shared by one of the authors of the work, 4 out of 10 were shared by the publishing journal itself or by some scientific institution, while the remaining 3 were shared by a single individual. This means that the social impact of the research did not emerge from the interest of Twitter users unaffiliated with the research, but rather from the very agents interested in its dissemination (primarily authors, publishers, or scientific institutions).

Based on all these pieces of evidence, it can be concluded that, at least in the field of using AI for emotions in education, the relevance of research to the scientific community and the corresponding impact on social media follow different paths. As demonstrated, there is a limited overlap between scientific relevance and social relevance, with the latter being largely maintained by the agents involved in the publications. If these findings were aligned with other fields of study, this evidence could serve as a starting point to raise awareness among the scientific community to seek different strategies, or to change current ones, in order to disseminate the results of their research more effectively and meaningfully on social media platforms. Without this translation of results beyond the scientific community, research has a low degree of transfer to society. Along the same lines, another implication of this study is that international accreditation agencies should analyze and reconsider the requirements for social impact in research in their various accreditation processes, due to the limited connection between both domains, scientific and social media, as showed in this research.

## 6 Limitations and prospective

In the analysis of the evolution of social impact records, only the data from Altmetric and Crossref have been considered, since PlumX does not provide temporal information about its records. Therefore, the evolution could be much greater if the records obtained from PlumX could be included.

Furthermore, the use of search strings exclusively in English excludes all research that does not have titles or abstracts in this language. The same applies to the choice of databases for obtaining the original sample, which in this case has been limited to Scopus and WOS, leaving out other databases that could expand the sample obtained.

Regarding prospective, based on the data obtained, other types of analysis can be conducted to deepen the relationship between scientific and social impact. For this purpose, a linear regression study can be designed to analyze the relationship between both types of impact.

In general terms, the proposed comparative methodology, which relates data obtained from a bibliometric study (along with its corresponding scientific impact from a scientometric perspective) with a descriptive analysis of the corresponding social impact, can be applied to any field of study. In this regard, it is worth noting that current trends in accreditation agencies propose the analysis of the impact of scientific research on social media (ANECA, 2024). Therefore, we consider it highly relevant to propose a methodology for this type of analysis.

## 7 Conclusion

The aim of the present research was to analyze the social impact of scientific production on the use of AI for emotions in the educational context. To achieve this, the PRISMA methodology was employed to obtain a sample, and based on this, Altmetric, Crossref, and PlumX databases were utilized to obtain records of social impact. All these data were processed using software developed specifically for this research, where a comparative study between bibliometric data of scientific production and its corresponding social impact was conducted.

The study focused on the evolution of scientific production with its corresponding social impact, sources, impact, and content analysis, revealing a high degree of social impact concerning scientific production, although social impact was primarily concentrated in 2022 (while scientific interest began in 2019) and the characteristics of social interactions were more superficial. This lack of alignment between the scientific dimension and the social impact of the field of study was also evident in the lack of coincidence between studies with more scientific impact regarding social impact, as well as in the different types of terms most used in both dimensions, a significant variability in the lag in months for scientific research to make an impact on social media, and the fact that the social impact of the research did not emerge from the interest of Twitter users unaffiliated with the research, but rather from the authors, publishers, or scientific institutions.

Overall, the aim was to highlight the importance of conducting studies on the social impact of scientific production, applicable to any field of study, and particularly relevant for analyzing the social impact of research in line with current trends of accreditation agencies.

## Data availability statement

The original contributions presented in the study are included in the article/supplementary material, further inquiries can be directed to the corresponding author.

## Author contributions

JR-S: Writing – original draft. SM-A: Writing – review & editing. AP: Writing – review & editing.

## Footnotes

^1^ http://www.plumanalytics.com

^2^ http://www.altmetric.com

^3^ http://www.crossref.org
